# FHL2 Silencing Reduces Wnt Signaling and Osteosarcoma Tumorigenesis *In Vitro* and *In Vivo*

**DOI:** 10.1371/journal.pone.0055034

**Published:** 2013-01-28

**Authors:** Julia Brun, François-Xavier Dieudonné, Caroline Marty, Judith Müller, Roland Schüle, Ana Patiño-García, Fernando Lecanda, Olivia Fromigué, Pierre J. Marie

**Affiliations:** 1 INSERM UMR 606, Paris, France; 2 Université Paris Diderot, Sorbonne Paris Cité, Paris, France; 3 Urologische Klink/Frauenklinik, Klinikum der Universität Freiburg and BIOSS Centre for Biological Signalling Studies, Freiburg, Germany; 4 Oncology Division, Center for Applied Medical Research, University of Navarra, Pamplona, Spain; Institut de Génomique Fonctionnelle de Lyon, France

## Abstract

**Background:**

The molecular mechanisms that are involved in the growth and invasiveness of osteosarcoma, an aggressive and invasive primary bone tumor, are not fully understood. The transcriptional co-factor FHL2 (four and a half LIM domains protein 2) acts as an oncoprotein or as a tumor suppressor depending on the tissue context. In this study, we investigated the role of FHL2 in tumorigenesis in osteosarcoma model.

**Methodology/Principal Findings:**

Western blot analyses showed that FHL2 is expressed above normal in most human and murine osteosarcoma cells. Tissue microarray analysis revealed that FHL2 protein expression is high in human osteosarcoma and correlates with osteosarcoma aggressiveness. In murine osteosarcoma cells, FHL2 silencing using shRNA decreased canonical Wnt/β-catenin signaling and reduced the expression of Wnt responsive genes as well as of the key Wnt molecules Wnt5a and Wnt10b. This effect resulted in inhibition of osteosarcoma cell proliferation, invasion and migration *in vitro*. Using xenograft experiments, we showed that FHL2 silencing markedly reduced tumor growth and lung metastasis occurence in mice. The anti-oncogenic effect of FHL2 silencing *in vivo* was associated with reduced cell proliferation and decreased Wnt signaling in the tumors.

**Conclusion/Significance:**

Our findings demonstrate that FHL2 acts as an oncogene in osteosarcoma cells and contributes to tumorigenesis through Wnt signaling. More importantly, FHL2 depletion greatly reduces tumor cell growth and metastasis, which raises the potential therapeutic interest of targeting FHL2 to efficiently impact primary bone tumors.

## Introduction

Osteosarcoma is the most common primary malignant bone tumor that occurs in children and young adults [1]. These tumors are characterized by a highly malignant and metastatic potential [2]. Despite aggressive chemotherapeutic treatment strategies, the rapid development of metastatic lesions and resistance to chemotherapy remain the major mechanisms responsible for the failure of treatments and poor survival rate of patients, which points to the need for new effective therapeutic strategies to prevent cell metastasis.

The molecular mechanisms that are involved in osteosarcoma growth and metastasis are not fully understood. A number of studies have suggested a role of Wnt signaling, an important pathway that controls osteoblastogenesis. Binding of canonical Wnts to frizzled (Fz) receptor and low-density lipoprotein 5 or 6 (LRP5/6) co-receptors leads to inhibition of β-catenin phosphorylation and subsequent translocation into the nucleus where it interacts with TCF/LEF transcription factors to activate the expression of Wnt-responsive genes [3]. Wnt signaling increases osteoprogenitor cell proliferation and their progression along the osteogenic lineage and prevents apoptosis in more mature osteoblasts [4], [5], [6]. A role of Wnt signaling in osteosarcoma development is supported by the finding that several Wnt ligands, receptors and co-receptors are highly expressed while Wnt inhibitors are downregulated in osteosarcoma cells [7]. It was also shown that the Wnt inhibitory factor 1 is epigenetically silenced in human osteosarcoma, and its disruption accelerates osteosarcoma development in mice [8]. Increased β-catenin-mediated activity has been frequently reported in osteosarcoma [9], [10], [11], further supporting a role for Wnt signaling in osteosarcoma development.

The transcriptional cofactor LIM-only protein FHL2 (four and a half LIM domains protein 2) is a multifunctional adaptor protein that is involved in the regulation of signal transduction, gene expression, cell proliferation and differentiation [12], [13]. The role of FHL2 in the development of cancers is complex. FHL2 was found to be down-regulated in some cancers and to be elevated in others compared to normal tissues, suggesting that FHL2 may act as an oncoprotein or a tumor suppressor, depending on its role as transcriptional activator or repressor in the cell type in which it is expressed [13]. One mechanism by which FHL2 may be linked to tumorigenesis is an interaction with key regulatory molecules. In muscle cells for example, FHL2 interacts with β-catenin and represses β-catenin-dependent transcription [14]. In contrast, in hepatoblastoma cells, FHL2 activates β-catenin-dependent transcription [15]. In bone, FHL2 was found to promote osteoblast differentiation [16], [17], [18]. We previously showed that FHL2 acts as an endogenous activator of mesenchymal cell differentiation into osteoblasts through its interaction with β-catenin and activation of Wnt/β-catenin signaling [19]. In these cells, overexpression of FHL2 increased Wnt/β-catenin signaling and osteogenic differentiation [19]. However, the implication of FHL2 in primary bone cancer progression and tumorigenesis has not been investigated.

In this study, we used a shRNA-based technique to study the contribution of FHL2 in primary bone tumor cell growth, invasion and migration, and we used xenograft experiments in mice to analyse the impact of FHL2 on tumorigenesis *in vivo*. Our data indicate that FHL2 silencing reduces osteosarcoma cell tumorigenesis *in vitro* and *in vivo*, indicating that FHL2 is a potential target for therapeutical intervention in this type of cancer.

## Results

### FHL2 Expression is Expressed Above Normal in Osteosarcoma

**Figure 1:**
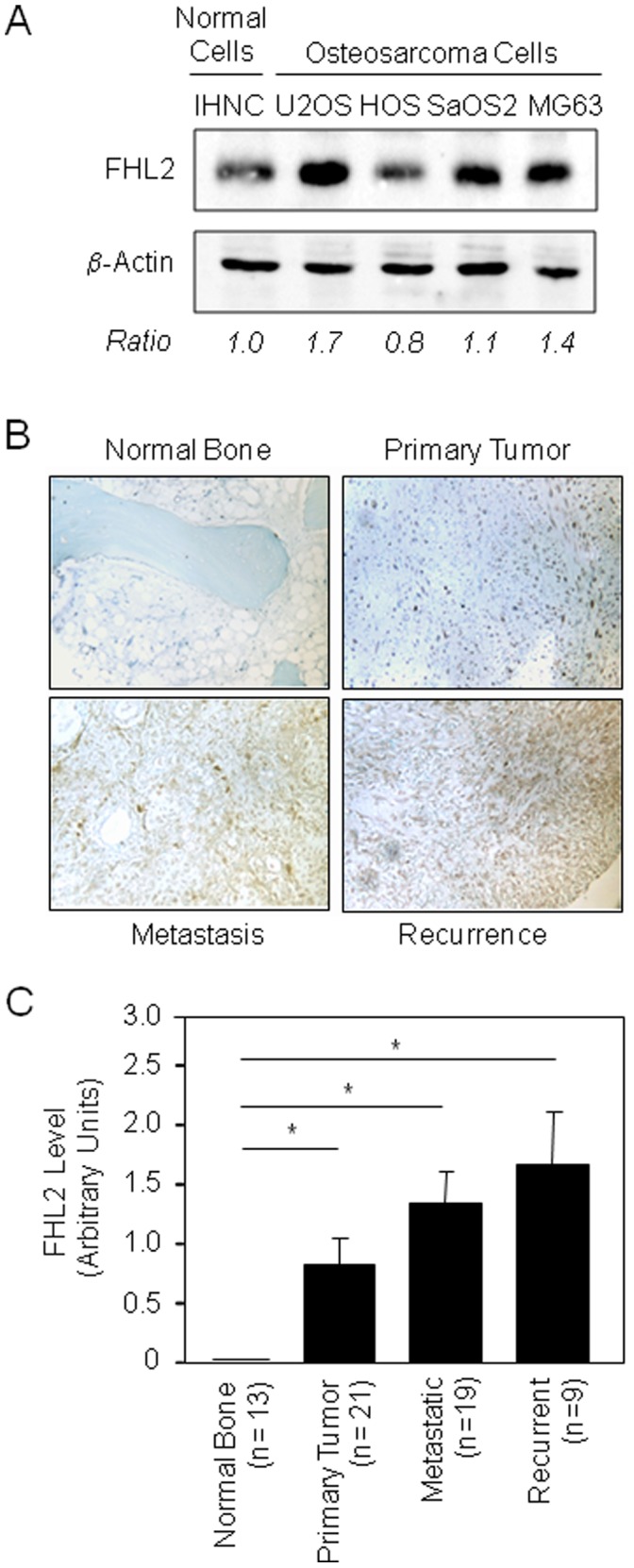
Basal FHL2 expression in human osteosarcoma cells and in tissue microarrays (TMA) of human osteosarcomas. Whole cell lysates were probed with the indicated antibody and revealed by Western blot analysis (A). FHL2 expression was determined by immunohistochemistry in tissue sections of normal bone, primary tumors, metastatic or recurrent osteosarcoma (Mag.×125) (B). Semi-quantitative scoring of immunohistochemical staining with anti-FHL2 antibody in normal bone and osteosarcoma samples according to patient outcome (primary tumor, metastatic or recurrent osteosarcoma) (C). *P<0.05.

**Figure 2:**
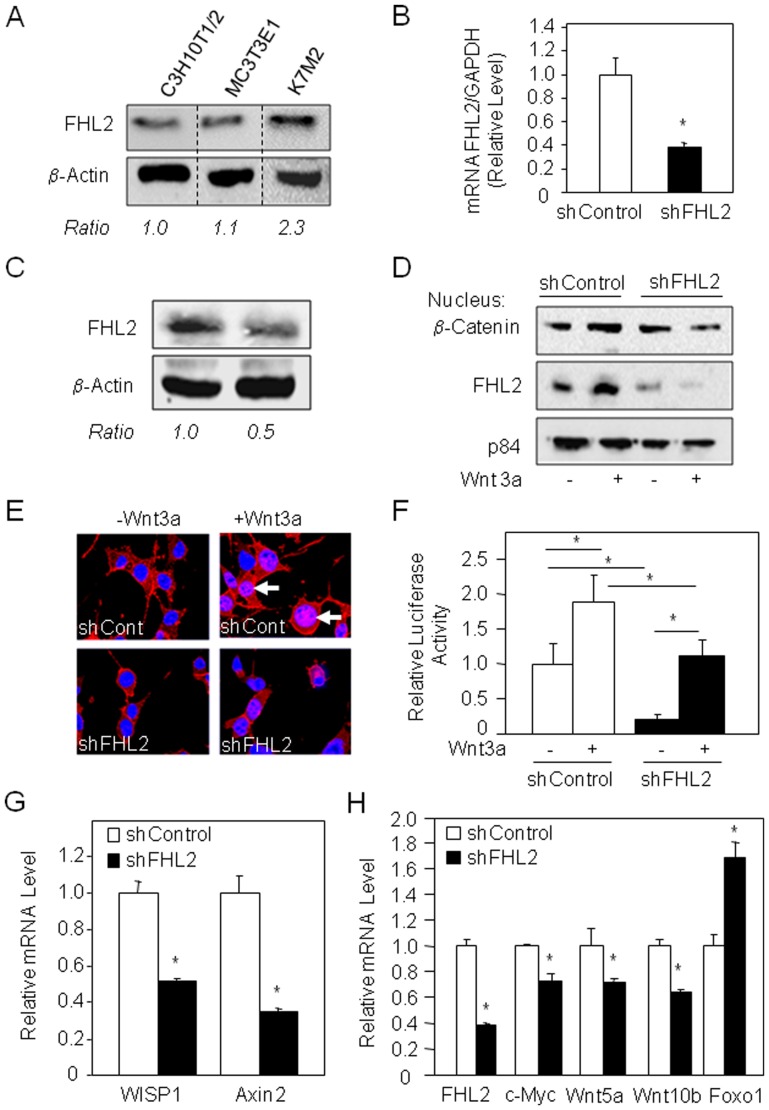
FHL2 silencing decreases Wnt/β-catenin signaling in osteosarcoma cells. Cell lysates of osteoblast precursor cell (C3H10T1/2), calvaria-derived osteoblastic cells (MC3T3E1) and osteosarcoma cell lines (K7M2) were analysed by western blot and FHL2 level was corrected for β-actin (A). After transduction with shControl or shFHL2, FHL2 levels in K7M2 cells were evaluated by q-PCR (B) and Western blot analysis (C). shControl and shFHL2 transduced K7M2 cells were treated for 24 h with Wnt3a CM and β-catenin nuclear translocation in K7M2 cells was evaluated by Western blot analysis of nuclear fraction (D), immunocytochemistry (red, arrows: β-catenin, blue: DAPI) (E), and β-catenin transcriptional activity was determined by a reporter assay (F). The mRNA levels in the shControl and shFHL2 cells were evaluated by q-PCR analysis (G, H). *: *P*<0.05 *vs* the indicated group or shControl cells.

We first analyzed by Western blot the expression of the FHL2 protein in a panel of human (U2OS, HOS, SaOS2, MG63) osteosarcoma cells with distinct genotypes compared to normal human osteoblasts (IHNC). We observed a single band at the predicted molecular weight in all cell lines tested (Fig. 1A). FHL2 protein level was slightly increased in SaOS2 cells compared to normal cells, and was robustly expressed in MG63 and U2OS osteosarcoma cells. These results support the concept that FHL2 is expressed above normal in some human osteosarcoma cells *in vitro*. To determine the potential role of FHL2 in human osteosarcoma, we investigated the expression of FHL2 in tissue microarray (TMA) from patients with osteosarcoma. Our immunohistochemical analysis showed that FHL2 was highly expressed in osteosarcoma tumors compared to normal bone (Fig. 1B). FHL2 expression tended to be higher in metastatic tumor cells compared to primary tumor cells (*P*<0.06). Furthermore, recurrent osteosarcoma tissues tended to exhibit the highest FHL2 level (*P*<0.07 vs metastatic cells). Semi-quantitative analysis indicated that the FHL2 protein expression increases with tumor grade in human osteosarcoma and correlates with osteosarcoma aggressiveness (Fig. 1C). To confirm this finding, we determined the expression of FHL2 in the aggressive and highly metastatic murine (K7M2) osteosarcoma cells [20]. We found that FHL2 protein level was 2-fold higher in K7M2 cells compared to normal murine C3H10T1/2 mesenchymal osteoprogenitors or to calvaria-derived MC3T3-E1 osteoblastic cells (Fig. 2A). Overall, these results suggest a role of FHL2 in osteosarcoma tumorigenesis.

### FHL2 Silencing Reduces Wnt/β-catenin Signaling in Osteosarcoma Cells

To investigate whether FHL2 may be a molecular target in bone cancer cells we used short hairpin RNA (shRNA)-mediated inhibition of FHL2 expression in the model of K7M2 osteosarcoma cells [20]. We found that shFHL2 transduction in K7M2 cells decreased FHL2 expression by 50–60% compared to control cells transduced with a non relevant shRNA, as shown by qPCR and western blot analyses (Fig. 2B, 2C). Using this tool, we examined the impact of shRNA-mediated inhibition of FHL2 expression on osteocarcoma cells behavior. We found that FHL2 silencing reduced β-catenin nuclear translocation induced by Wnt3a in K7M2 cells, as shown by Western blot analysis (Fig. 2D), immunocytochemistry (Fig. 2E), and the reduced β-catenin transcriptional activity in the presence or absence of Wnt3a (Fig. 2F). To confirm the impact of FHL2 silencing on Wnt signaling in osteosarcoma cells, we performed a molecular analysis of Wnt responsive gene expression. We found that FHL2 silencing in K7M2 cells strongly decreased the expression of Axin2 and WISP-1 which are direct Wnt target genes [21] (Fig. 2G). FHL2 silencing also decreased the expression of c-Myc, which is involved in cell proliferation, and Wnt5a and Wnt10b, which are involved in osteosarcoma severity and invasiveness [22], [23], [24]. Furthermore, FHL2 silencing increased the expression of the Forkhead class box protein O transcription factor 1 (Foxo1), which is transcriptionally activated by β-catenin [25] (Fig. 2H). Overall, these results indicate that FHL2 silencing reduces β-catenin signaling and Wnt-responsive gene expression in murine osteosarcoma cells.

### FHL2 Silencing Reduces Cell Proliferation and Apoptosis in Osteosarcoma Cells

**Figure 3:**
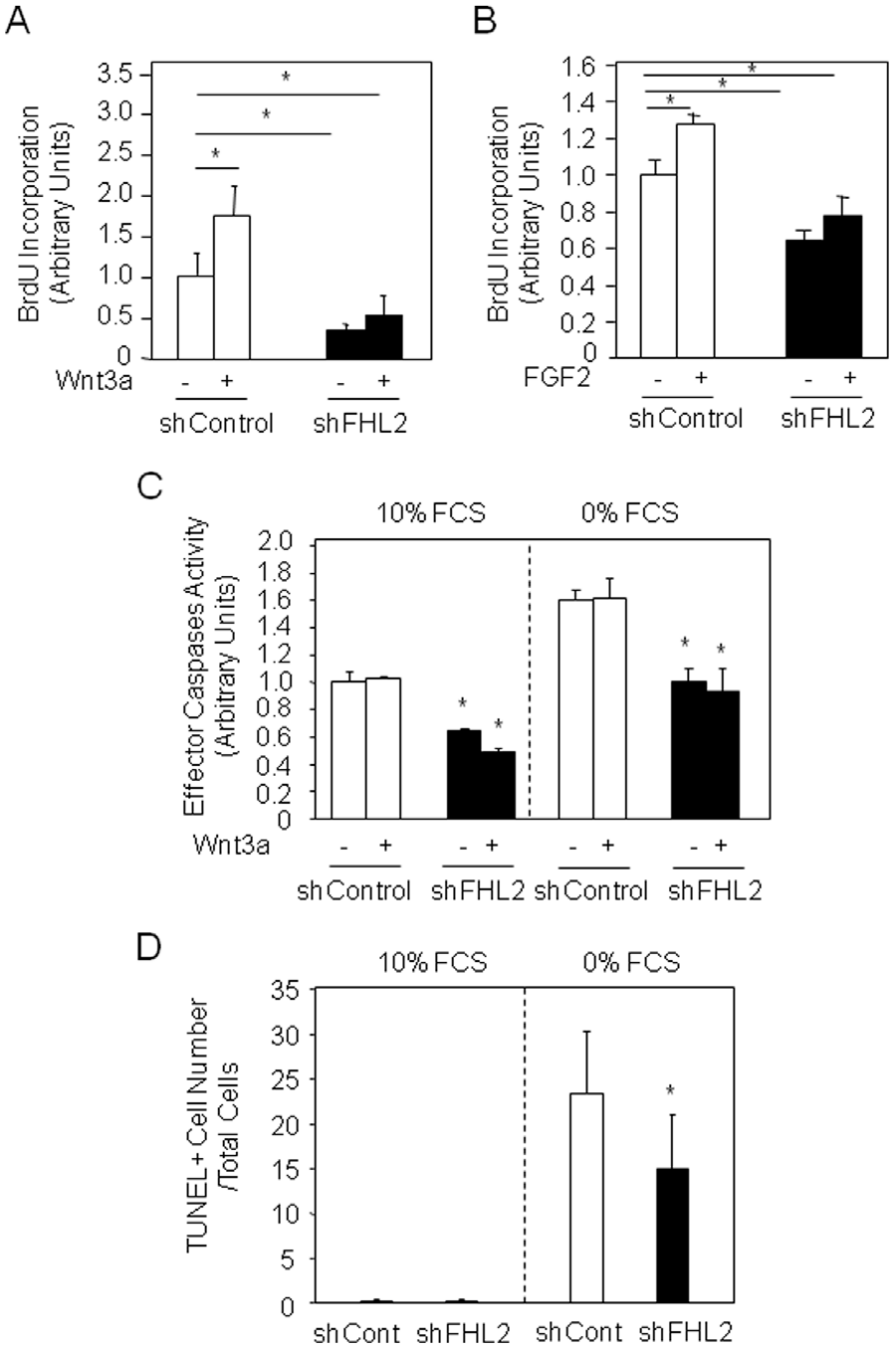
FHL2 silencing decreases osteosarcoma cell growth. After treatment with Wnt3a CM (A) or FGF-2 (0.50 ng/ml**)** (B) for 3 days, DNA replication was evaluated by BrdU incorporation in shControl and shFHL2-transduced K7M2 cells. Apoptosis was induced by serum deprivation and effector caspases activity was evaluated at 48 h in cells treated with or without Wnt3a CM (C). a: p<0.05 *vs* untreated, b:p<0.05 *vs* shControl. TUNEL analysis was performed in basal and serum deprivation conditions at 72 h (D). *: *P*<0.05 *vs* the indicated group or shControl cells.

We next determined the consequences of FHL2 silencing on osteosarcoma cell growth and survival in basal and Wnt3a-supplemented medium. As shown in Fig. 3A, Wnt3a supplementation increased cell replication in K7M2 cells, as determined by the BrdU incorporation assay. Silencing FHL2 resulted in decreased cell proliferation in basal conditions (Fig. 3A). Furthermore, FHL2 silencing abolished the stimulatory effect of Wnt3a on cell proliferation (Fig. 3A). However, the mitogenic effect of FGF-2 (0.50 ng/ml) was also abrogated by shFHL2 (Fig. 3B), suggesting that FHL2 silencing has a general inhibitory effect on cell proliferation. We next examined whether FHL2 silencing may affect osteosarcoma cell death. We found that FHL2 silencing reduced effector caspases activity in murine K7M2 osteosarcoma cells in basal and serum-deprived conditions (Fig. 3C). Importantly, the same inhibitory effect was found in the presence of Wnt3a which had no effect on caspases activity in these cells (Fig. 3C). These results indicate that the decreased caspases activity induced by FHL2 silencing occurred independently of Wnt3a signaling. Although FHL2 silencing reduced caspases activity in K7M2 cells, this effect had limited impact on cell death as the number of apoptotic cells was only slightly decreased, as shown by TUNEL analysis (Fig. 3D). Overall, these data suggest that FHL2 silencing slightly impacts K7M2 proliferation and apoptosis through mechanisms independent of Wnt3a.

### FHL2 Silencing Reduces Cell Invasion and Migration *in vitro*

**Figure 4:**
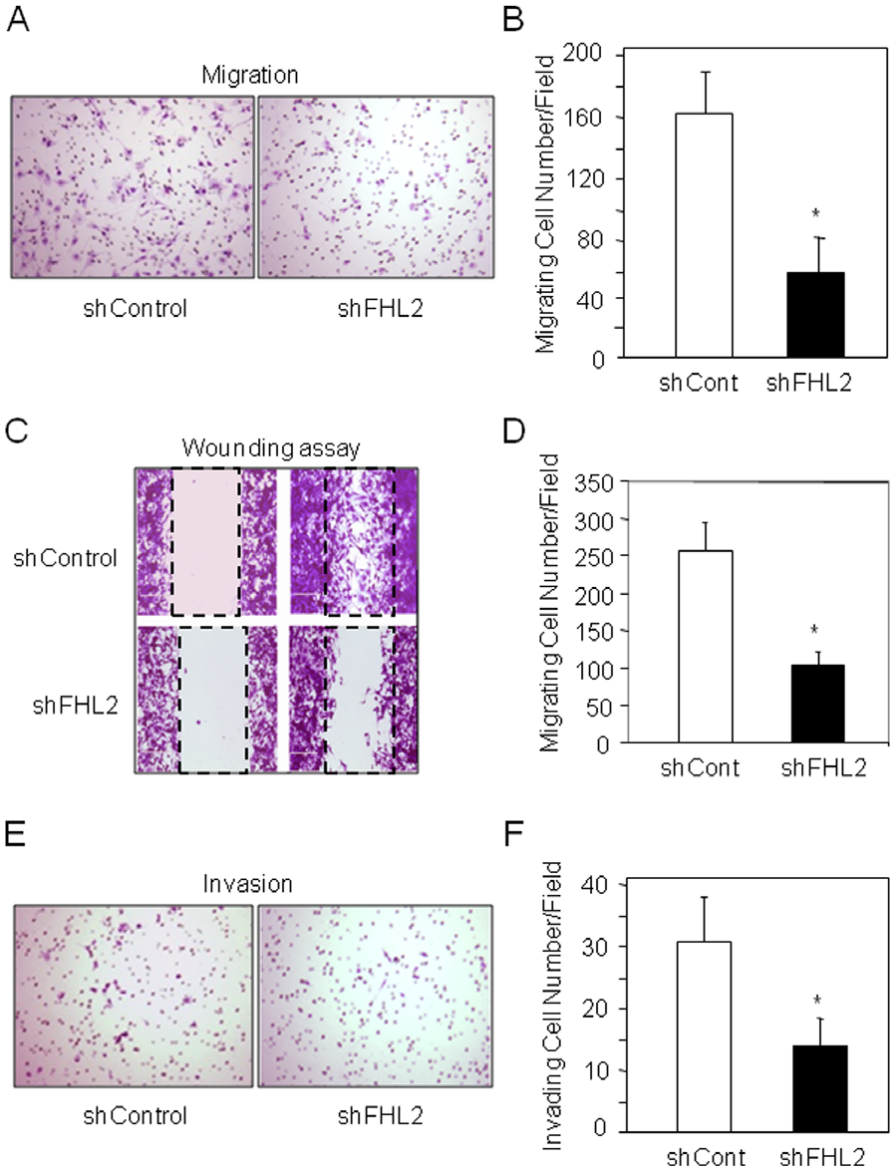
FHL2 silencing decreases bone tumor cell migration and invasion. Migration of shControl and shFHL2-transduced K7M2 cells was evaluated by Boyden’s chamber (A) and wounding assays (C) and migrating cell number was evaluated (B, D). K7M2 cell invasion was evaluated by the Matrigel invasion assay (E, F). *: *P*<0.05 *vs* shControl-transduced cells.

Because the development of metastasis is highly dependent on cell migration and invasion [26], we investigated the impact of FHL2 silencing on the invasiveness potential of the highly metastatic K7M2 osteosarcoma cells [27]. Strikingly, silencing FHL2 markedly reduced cell migration compared to control cells (Fig. 4A, B). In direct support of this finding, FHL2 silencing in K7M2 cells markedly decreased cell wounding compared to control cells (Fig. 4C, D). Given the large impact of FHL2 silencing on K7M2 migration, we analyzed whether FHL2 silencing may also reduce bone tumor cell invasion. We found that Matrigel invasion was markedly reduced in shFHL2 transduced K7M2 cells compared to control cells (Fig. 4E, F). Taken together, these data show that silencing FHL2 reduces murine tumor cell invasion and migration *in vitro*.

### FHL2 Silencing Reduces Tumorigenesis and Metastasis *in vivo*

**Figure 5:**
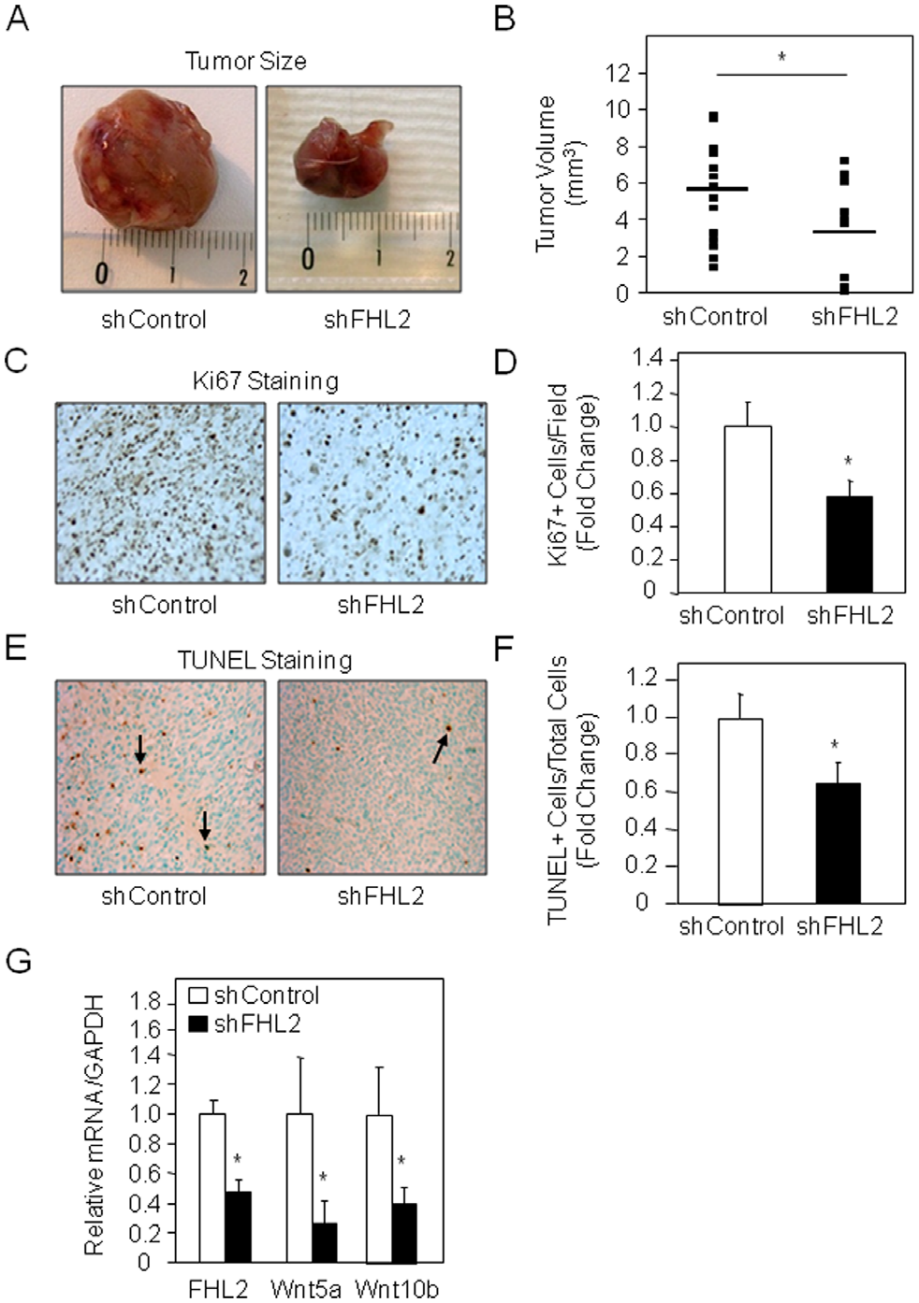
FHL2 silencing decreases bone tumor growth *in vivo*. shRNA control and shFHL2-transduced murine K7M2 cells were injected in BALB/c mice and tumor size (A) and volume (B) were determined at 6 weeks (n = 9 per group). Cell proliferation and apoptosis in tumors was determined by histological analysis using Ki67 (C, D) and TUNEL staining (arrows), respectively (E, F). Wnt5a and Wnt10b mRNA expression was evaluated in the tumors by q-PCR analysis (G). *: *P*<0.05 *vs* shControl cells.

Based on the above evidence that FHL2 silencing reduces mouse osteosarcoma cell migration and invasiveness *in vitro*, we hypothesized that this effect may impact osteosarcoma tumorigenesis *in vivo*. To investigate this hypothesis, shControl- and shFHL2-transduced K7M2 cells were injected in thigh muscle of BALB/c mice. As expected, injected K7M2 cells developed large tumors which were detectable after 6 weeks (Fig. 5A). We found that FHL2 silencing strikingly reduced tumor size compared to control cells (Fig. 5A). Quantification of the tumor samples confirmed that FHL2 silencing reduced tumor volume by about 2-fold compared to control tumors (Fig. 5B), which is consistent with the anticancer activity of FHL2 silencing that we found *in vitro*.

Osteosarcoma development arises in large part from deregulated cell growth [28]. We therefore investigated whether the inhibition of tumor growth induced by FHL2 silencing is related to decreased cancer cell replication. Analysis of cell replication using Ki67 immunostaining showed that FHL2 silencing decreased the number of Ki67-positive cells (Fig. 5C). Quantification revealed that cell replication was reduced by about 40% in the tumor (Fig. 5D). We also analyzed the effect of FHL2 silencing on osteosarcoma cell death using TUNEL analysis. Consistent with our *in vitro* data we found reduced apoptosis in tumors derived from shFHL2-infected K7M2 cells compared to tumors derived from control cells (Fig. 5E, F). These data indicate that shRNA-targeted FHL2 expression reduced tumor growth through a decreased cell replication and despite a slight reduction of apoptosis in murine osteosarcoma cells. We next analysed whether FHL2 silencing impacted Wnt responsive genes, as found *in vitro* (Fig. 2H). As shown in Fig. 5G, a quantitative PCR analysis of RNA isolated from the tumors revealed that FHL2 silencing markedly reduced Wnt5a and Wnt10b mRNA level of expression. These results indicate that FHL2 silencing reduces Wnt family proteins expression and impacts Wnt signaling in murine osteosarcoma tumors *in vivo.*

**Figure 6:**
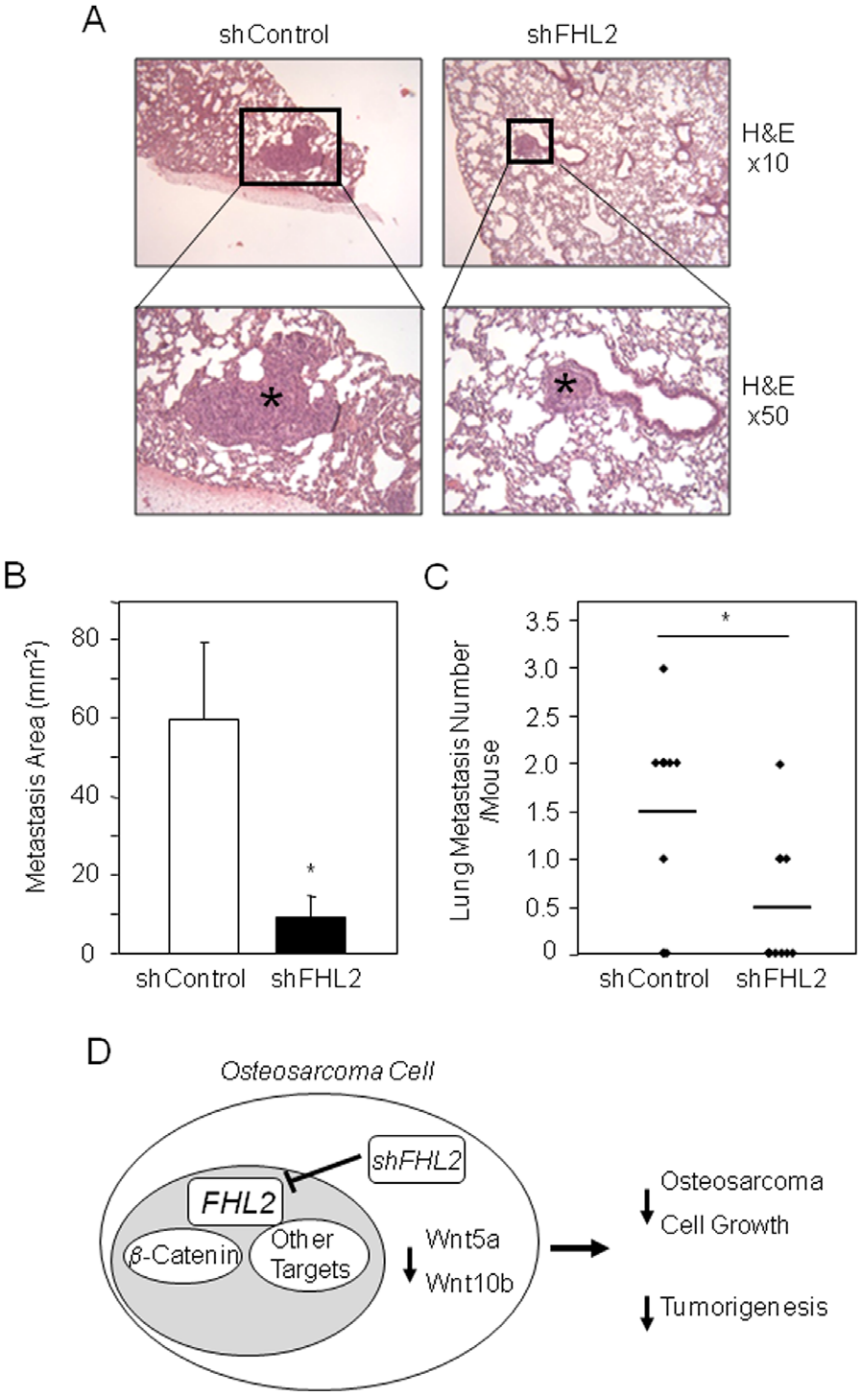
FHL2 silencing reduces lung metastasis in mice. Histological hematoxylin/eosin (H&E) staining of lung tissue sections showing metastasis (stars) developed in mice injected IM with shControl or shFHL2-transduced K7M2 cells (A). Metastasis area (B) and number (C) in the lung tissue were evaluated. Results are expressed as mean ± s.d. (n = 9 animals per group). *P<0.05 *vs* shControl. Proposed model in which FHL2 silencing using shFHL2 in murine osteosarcoma cells attenuates Wnt/β-catenin signaling and reduces the expression of Wnt5a and Wnt10b and possibly other FHL2 target genes in the tumors, resulting in decreased osteosarcoma cell growth, invasiveness and tumorigenesis *in vivo* (D).

Because lung metastasis is a major clinical issue in osteosarcoma, we investigated whether FHL2 silencing may impact osteosarcoma cell invasiveness in mice. As shown in Fig. 6A, mice injected with shFHL2-infected K7M2 cells developed less lung metastasis than mice injected with shControl-K7M2 cells. Both the number and the surface of the lung metastasis were markedly reduced by FHL2 silencing (Fig. 6 B, C). Overall, the data indicate that FHL2 is overexpressed in osteosarcoma and demonstrate that silencing FHL2 reduces Wnt signaling and decrease osteosarcoma cell growth, invasiveness and tumorigenesis *in vivo* (Fig. 6D).

## Discussion

In this study, we determined the role of the multifunctional protein FHL2 in primary bone cancer growth and tumorigenesis *in vitro* and *in vivo*. We first investigated whether FHL2 expression is deregulated in bone tumor cells. Our data indicate that FHL2 is expressed above normal in several human osteosarcoma cell lines and in the aggressive K7M2 murine osteosarcoma cells. Other studies have reported variable FHL2 gene expression in human soft tissue cancers, depending on the cell type. Notably, FHL2 was found to be increased in breast cancer [29], glioma [30], lung cancer [31], colon carcinoma [32] and gastrointestinal cancer [33] compared to normal tissues. In contrast, FHL2 was found to be down-regulated in rhabdomyosarcomas [14] and in prostate cancer [34]. The variable expression of FHL2 in cancer cells is likely related to its distinct roles depending on the cell type in relation with FHL2 interaction with other proteins, causing either repression or activation of target genes [13]. The present finding that FHL2 protein level is high in osteosarcoma tumors and correlates with osteosarcoma aggressiveness in human osteosarcoma supports a positive role of FHL2 in bone tumor development.

To investigate the specific role of FHL2 in osteosarcoma tumor development, we used K7M2 murine osteosarcoma cells that express high FHL2 levels in basal conditions. We found that silencing FHL2 though transduction with a lentivirus encoding a specific shRNA that efficiently reduced FHL2 levels in these cells, reduced cell proliferation and repressed the oncogene c-Myc, supporting a role of FHL2 in osteosarcoma cell growth. This is consistent with the recent observation that FHL2 deficiency reduces intestinal tumorigenesis in Apc mutant mice [32]. Several mechanisms such as the androgen receptor [35], partners of the MAPK pathway [36], [37], [38], Smad proteins [39] and cyclin D1 [40] have been reported to be involved in the control of cell replication and the response to mitogenic stimulation by FHL2. We focused here on Wnt/β-catenin signaling since FHL2 interacts with this pathway in several cell types [14], [15], [41] including in osteoblast progenitor cells [19]. Additionally, Wnt/β-catenin signaling was reported to be dysregulated in osteosarcoma [7], [8], [9], [11]. We found that FHL2 silencing in murine osteosarcoma cells led to reduce β-catenin transcription as well as the expression of c-myc, a target of Wnt/β-catenin signaling which controls cell replication, and concomitantly reduced the expression of Axin2 and WISP-1 which are direct Wnt/β-catenin-target genes [3]. Although FHL2 silencing abrogated the positive effect of Wnt3a on osteosarcoma cell growth, this effect might be explained by overall reduced proliferation since FHL2 silencing also abrogated FGF-2-induced cell proliferation. We also found that FHL2 silencing slightly reduced osteosarcoma cell apoptosis *in vitro*. To date, both pro-apoptotic and anti-apoptotic effects of FHL2 have been reported [42], [43]. This dual effect is likely to be related to the cellular context, namely the molecular interactions between FHL2 and specific partners [13]. In the present study, FHL2 silencing may have inhibited cell proliferation independently of cell death since FHL2 was found to regulate tumor cell growth through the control of G1/S transition during cell cycle rather than apoptosis [43]. One possibility is that the depletion of FHL2 may have resulted in cells becoming more quiescent, thus avoiding cell cycle-related initiation of apoptosis. The observed anti-apoptotic effect of FHL2 silencing in osteosarcoma cells may be linked in part to the observed decrease in Wnt5a, since this protein exerts anti-apoptotic activity in cells of the osteoblast lineage [44]. FHL2 is known to interact with Foxo1 [45] and Foxo1 was shown to increase osteoblast apoptosis *in vivo* [46]. We found that FHL2 silencing increased Foxo1 expression in osteosarcoma cells, suggesting a possible implication of Foxo1 in the anti-apoptotic effect of FHL2 silencing in osteosarcoma cells. Despite our finding that FHL2 silencing reduced osteosarcoma cell apoptosis *in vitro* and *in vivo*, we found that the overall effect of FHL2 silencing *in vivo* is to suppress tumor growth, indicating that FHL2 acts mostly as an oncoprotein in osteosarcoma cells.

Osteosarcoma tumorigenesis is often associated with tumor cell invasion leading to metastasis and reduced patient’s survival [1], [26]. Few experimental studies suggest that FHL2 may play a role in cancer cell invasion and migration in some soft tissue cancers [32], [47], [48]. However, nothing is known on the role of FHL2 in osteosarcoma cell metastasis capacity. Strikingly, we found that FHL2 silencing reduced osteosarcoma cell invasion and migration *in vitro* and metastatic development *in vivo*. These results provide the first evidence that FHL2 is involved in the invasiveness capacity of osteosarcoma cells and that silencing FHL2 reduces osteosarcoma tumorigenesis in mice. One mechanism underlying the anti-oncogenic effect of FHL2 silencing could be the decreased expression of the Wnt family members Wnt5a and Wnt10b that we observed *in vitro* and *in vivo,* because these proteins confer cell invasiveness, metastasis and reduced survival in osteosarcomas [22], [23], [24] and thereby contribute to tumorigenesis [49], [50]. In addition to involve Wnt proteins, the anti-oncogenic effect of FHL2 silencing may involve decreased interaction of FHL2 with integrins [18], [51] which are also critical for cancer cell adhesion to extracellular matrix, migration and invasion.

In summary, we show here for the first time that the expression of the Wnt co-regulator FHL2 is high in invasive osteosarcoma and that FHL2 acts as an oncoprotein in osteosarcoma cells. More importantly, we demonstrate that silencing FHL2 represses osteosarcoma cell growth and tumorigenesis *in vitro* and *in vivo*. Overall, the data indicate that targeting FHL2, a Wnt activator in osteosarcoma cells, may be useful for therapeutical intervention in this type of cancer.

## Materials and Methods

### Cell Culture and Transduction

The cancer cells derived from different osteosarcoma tumors used were p53-deficient SaOS2 human cells, p53 mutant MG63 human cells, HOS and U2OS human cells, and K7M2 murine osteosarcoma cells [27], all obtained from ATCC (Rockville, MD, USA). Normal human osteoblasts (IHNC) were obtained from human neonatal calvaria, and murine C3H10T1/2 and MC3T3-E1 cells were from ATCC. The cells were cultured in DMEM (Invitrogen Corporation, Paisley, Scotland) in the presence of 10% heat inactivated FCS, 1% L-glutamine and penicillin/streptomycin (10,000 U/ml and 10,000 µg/ml, respectively) with medium change every 2–3 days. For FHL2 silencing, lentiviral particules containing shRNA directed against mouse FHL2 or a control shRNA that does not recognize mouse FHL2 were used according to the manufacturer recommendations (Santa Cruz Biotechnology, CA, USA).

### Cell Proliferation Assay

For cell proliferation assay, K7M2 cells were seeded at 3×10^3^ cells/cm^2^ and cell number was evaluated by cell counting. DNA replication was evaluated using a BrdU ELISA assay (GE Healthcare, Buckinghamshire, UK) as previously described [52]. Cells were treated with Wnt3a conditioned medium (CM) obtained as described previously [19] or human recombinant FGF-2 (Peprotech Neuilly-Sur-Seine, France) at the indicated time point.

### Cell Death Assays

DNA fragmentation was detected using TUNEL staining and effector caspases activity was determined using Ac-DEVD-pNA as substrate (Alexis Biochemicals, CA, USA) [53].

### Cell Invasion and Migration Assays

Wounding assay was performed according to the manufacturer’s instructions (Ibidi, BioValley, Marne la Vallée, France). Recovery of the denuded area was computerized using an inverted microscope (Leica, Cambridge, UK). Cell migration and invasion were determined in the modified Boyden’s chamber assay, as described previously [52].

### β-catenin Reporter Assay

β-catenin transcriptional activity was determined by Firefly and Renilla luciferase assays using a Luciferase Reporter Assay System according to the manufacturer’s recommendations (Promega, Charbonnieres, France).

### RT-qPCR Analysis

Total RNA was isolated using Trizol Reagent (Eurobio Laboratories, Les Ulis, France) according to the manufacturer’s instructions. Three µg of total RNA from each samples were reverse transcribed with 1× RT buffer, 1 mM dNTP mix, 1× random primers and 50 U multiscribe reverse transcriptase (Applied Biosystems, Villebon sur Yvette, France) in a total volume of 20 µl, at 37°C for 2 h. The relative mRNA levels were evaluated by quantitative RT-PCR using LightCycler Instrument (Roche Applied Science, Indianapolis Ind., USA) and SYBR Green PCR kit (ABGen, Courtabœuf, France). GAPDH was used as internal control. Primers were as follow: c-Myc forward 5′-CGGTTTCTCAGCCGCTGCCA-3′ and reverse 5′-TGGGCGAGCTGCTGTGCTTG-3′; Wnt5a forward 5′-CCCCGACGCTTCGCTTGAATTCC-3′ and reverse 5′-CCCAAAGCCACTCCCGGGCTTAA-3′; Wnt10b forward 5′-CCGGGACATCCAGGCGAGAA-3′ and reverse 5′-AGCTGCCTGACGTTCCATGGC-3′; Foxo1 forward 5′-AGATGAGTGCCCTGGGCAGC-3′ and reverse 5′-GATGGACTCCATGTCAACAGT-3′; FHL2 forward 5′-TGCGTGCAGTGCAAAAAG-3′ and reverse 5′-TGTGCACACAAAGCATTCCT-3′; GAPGH forward 5′-ACACATTGGGGGTAGGAACA-3′ and reverse 5′-AACTTTGGCATTGTGGAAGG-3′; Axin 2 forward 5′-GAGAGTGAGCGGCAGAGC-3′ and reverse 5′-CGGCTGACTCGTTCTCCT-3′; WISP1 forward 5′-TGGACATCCAACTACACATCAA-3′ and reverse 5′-AAGTTCGTGGCCTCCTCTG-3′.

### Immunoblot Analysis

Cell lysates were prepared and resolved on 10% SDS-PAGE as previously described [19] were incubated with rabbit anti-FHL2 (1/1000; Abcam, Cambridge, UK), mouse anti-β-catenin (1/1000; Santa Cruz, Santa Cruz Biotechnology, CA, USA), rabbit anti-β-actin (1/2000; Sigma-Aldrich, St Quentin Fallavier, France) or mouse anti-p84 (1/1000; Abcam) antibodies. Membranes were then incubated with appropriate HRP-conjugated secondary antibody (1/20,000). The signals were visualized with enhanced chemiluminescence western blotting detection reagent (Immun-star chemiluminescent kit, BioRad, Marnes-la-Coquette, France) and autoradiographic film (X-OMAT-AR, Eastman Kodak Company, Rochester, NY, USA). Densitometric analysis using QuantityOne software (BioRad) was performed following digital scanning (Agfa, Japan). Representative images of immunoblots are shown.

### Immunocytochemistry

For immunocytochemistry, cells were fixed with 4% PFA in PBS for 10 min at 4°C, washed twice with PBS, permeabilized with 0.025% Triton X-100 for 5 min and blocked with 3% BSA in PBS for 15 minutes at room temperature. Cells were incubated overnight at 4°C with anti-β-catenin antibody (Santa Cruz) used at 1∶100 dilution, then incubated with a secondary antibody (goat anti-rabbit conjugated to Cy3; Beckman Coulter, Villepinte, France). Cover glasses were viewed using apotome fluorescence microscopy (Carl Zeiss, Jena, Germany).

### Human Tissue Microarray

Tissue microarray (TMA) composed of paraffin-embedded 231 tissue cores were deparaffinized and rehydrated. Antigen retrieval was performed using citrate buffer (ph 6) at 70°C during 4 h followed by permeabilisation with saponin (0.1%) for 30 min, before incubation with polyclonal anti-FHL2 antibody [54] used at 1∶300 overnight at 4°C. The signal was revealed using Vectastain Elite ABC system (Vector Laboratories Ltd, Peterborough, UK) and estimated without prior information about the TMA spots.

### Murine Tumor and Metastatic Models

This study was carried out in strict accordance with the recommendations in the Guide for the Care and Use of Laboratory Animals of the Institut National de la Santé et de la Recherche Médicale. The protocol was approved by the Committee on the Ethics of Animal Experiments of Lariboisière-Villemin (Permit Number: CEEALV/2011-01-05). We used K7M2 cells that are aggressive mouse osteosarcoma cells that form tumors and spontaneously metastasize following injection. Female BALB/c mice (4-weeks old; Harlan, Gannat, France) were intramuscularly injected with 10^6^ cells/20 µl of PBS in thigh muscles (one per leg; 9 mice per group). After 6 weeks, mice were euthanized, all tumors were dissected, and tumor size was determined using a calliper. Primary tumors and lungs were fixed in formalin and included in paraffin. Tissue sections (5 µm) were stained with hematoxylin/eosin or immunostained with anti-Ki67 antibody (1/100; Abcam). All fields located outside of the necrotic center and without the remaining muscular fibers were microphotographed under an Olympus microscope. TUNEL assay was performed using the Apoptag® Peroxidase In Situ Apoptosis Detection Kit (Millipore, Billerica, MA, USA) according to the manufacturer’s recommendations.

### Statistical Analysis

The *in vitro* data are the mean ± s.d. and are representative of at least three experiments. The *in vivo* data are the mean ± s.d. The data were analyzed by Student’s t-test with *P*<0.05 considered to be significant. The TMA scoring was expressed as the mean ± s.e.m. and was analysed by Kruskal-Wallis test followed by Tukey test.

## References

[1] Himelstein BP (1998) Osteosarcoma and other bone cancers. Curr Opin Oncol 10: 326–333. 9702400 10.1097/00001622-199807000-00009

[2] Bruland OS, Hoifodt H, Saeter G, Smeland S, Fodstad O (2005) Hematogenous micrometastases in osteosarcoma patients. Clin Cancer Res 11: 4666–4673. 16000559 10.1158/1078-0432.CCR-05-0165

[3] Nusse R (2005) Wnt signaling in disease and in development. Cell Res 15: 28–32. 15686623 10.1038/sj.cr.7290260

[4] Bodine PV, Komm BS (2006) Wnt signaling and osteoblastogenesis. Rev Endocr Metab Disord 7: 33–39. 16960757 10.1007/s11154-006-9002-4

[5] Bodine PV (2008) Wnt signaling control of bone cell apoptosis. Cell Res 18: 248–253. 18212734 10.1038/cr.2008.13

[6] Gaur T, Lengner CJ, Hovhannisyan H, Bhat RA, Bodine PV, et al (2005) Canonical WNT signaling promotes osteogenesis by directly stimulating Runx2 gene expression. J Biol Chem 280: 33132–33140. 16043491 10.1074/jbc.M500608200

[7] McQueen P, Ghaffar S, Guo Y, Rubin EM, Zi X, et al (2011) The Wnt signaling pathway: implications for therapy in osteosarcoma. Expert Rev Anticancer Ther 11: 1223–1232. 21916576 10.1586/era.11.94

[8] Kansara M, Tsang M, Kodjabachian L, Sims NA, Trivett MK, et al (2009) Wnt inhibitory factor 1 is epigenetically silenced in human osteosarcoma, and targeted disruption accelerates osteosarcomagenesis in mice. J Clin Invest 119: 837–851. 19307728 10.1172/JCI37175 PMC2662557

[9] Haydon RC, Deyrup A, Ishikawa A, Heck R, Jiang W, et al (2002) Cytoplasmic and/or nuclear accumulation of the beta-catenin protein is a frequent event in human osteosarcoma. Int J Cancer 102: 338–342. 12402302 10.1002/ijc.10719 PMC4122310

[10] Entz-Werle N, Lavaux T, Metzger N, Stoetzel C, Lasthaus C, et al (2007) Involvement of MET/TWIST/APC combination or the potential role of ossification factors in pediatric high-grade osteosarcoma oncogenesis. Neoplasia 9: 678–688. 17786187 10.1593/neo.07367 PMC1950438

[11] Dieudonné FX, Marion A, Haÿ E, Marie PJ, Modrowski D (2010) High Wnt signaling represses the proapoptotic proteoglycan syndecan-2 in osteosarcoma cells. Cancer Res 70: 5399–5408. 20530678 10.1158/0008-5472.CAN-10-0090

[12] Johannessen M, Moller S, Hansen T, Moens U, Van Ghelue M (2006) The multifunctional roles of the four-and-a-half-LIM only protein FHL2. Cell Mol Life Sci 63: 268–284. 16389449 10.1007/s00018-005-5438-z PMC11136317

[13] Kleiber K, Strebhardt K, Martin BT (2007) The biological relevance of FHL2 in tumour cells and its role as a putative cancer target. Anticancer Res 27: 55–61. 17352216

[14] Martin B, Schneider R, Janetzky S, Waibler Z, Pandur P, et al (2002) The LIM-only protein FHL2 interacts with beta-catenin and promotes differentiation of mouse myoblasts. J Cell Biol 159: 113–122. 12370240 10.1083/jcb.200202075 PMC2173499

[15] Wei Y, Renard CA, Labalette C, Wu Y, Levy L, et al (2003) Identification of the LIM protein FHL2 as a coactivator of beta-catenin. J Biol Chem 278: 5188–5194. 12466281 10.1074/jbc.M207216200

[16] Amaar YG, Thompson GR, Linkhart TA, Chen ST, Baylink DJ, et al (2002) Insulin-like growth factor-binding protein 5 (IGFBP-5) interacts with a four and a half LIM protein 2 (FHL2). J Biol Chem 277: 12053–12060. 11821401 10.1074/jbc.M110872200

[17] Gunther T, Poli C, Muller JM, Catala-Lehnen P, Schinke T, et al (2005) Fhl2 deficiency results in osteopenia due to decreased activity of osteoblasts. Embo J 24: 3049–3056. 16079911 10.1038/sj.emboj.7600773 PMC1201354

[18] Lai CF, Bai S, Uthgenannt BA, Halstead LR, McLoughlin P, et al (2006) Four and half lim protein 2 (FHL2) stimulates osteoblast differentiation. J Bone Miner Res 21: 17–28. 16355270 10.1359/JBMR.050915

[19] Hamidouche Z, Haÿ E, Vaudin P, Charbord P, Schüle R, et al (2008) FHL2 mediates dexamethasone-induced mesenchymal cell differentiation into osteoblasts by activating Wnt/beta-catenin signaling-dependent Runx2 expression. FASEB J 22: 3813–3822. 18653765 10.1096/fj.08-106302

[20] Khanna C, Khan J, Nguyen P, Prehn J, Caylor J, et al (2001) Metastasis-associated differences in gene expression in a murine model of osteosarcoma. Cancer Res 61: 3750–3759. 11325848

[21] He TC, Sparks AB, Rago C, Hermeking H, Zawel L, et al (1998) Identification of c-MYC as a target of the APC pathway. Science 281: 1509–1512. 9727977 10.1126/science.281.5382.1509

[22] Chen K, Fallen S, Abaan HO, Hayran M, Gonzalez C, et al (2008) Wnt10b induces chemotaxis of osteosarcoma and correlates with reduced survival. Pediatr Blood Cancer 51: 349–355. 18465804 10.1002/pbc.21595

[23] Enomoto M, Hayakawa S, Itsukushima S, Ren DY, Matsuo M, et al (2009) Autonomous regulation of osteosarcoma cell invasiveness by Wnt5a/Ror2 signaling. Oncogene 28: 3197–3208. 19561643 10.1038/onc.2009.175

[24] Lu BJ, Wang YQ, Wei XJ, Rong LQ, Wei D, et al (2012) Expression of WNT-5a and ROR2 correlates with disease severity in osteosarcoma. Mol Med Report 5: 1033–1036. 10.3892/mmr.2012.772 PMC3493076 22293903

[25] Essers MA, de Vries-Smits LM, Barker N, Polderman PE, Burgering BM, et al (2005) Functional interaction between beta-catenin and FOXO in oxidative stress signaling. Science 308: 1181–1184. 15905404 10.1126/science.1109083

[26] Jaffe N (2009) Osteosarcoma: review of the past, impact on the future. The American experience. Cancer Treat Res 152: 239–262. 20213394 10.1007/978-1-4419-0284-9_12

[27] Khanna C, Prehn J, Yeung C, Caylor J, Tsokos M, et al (2000) An orthotopic model of murine osteosarcoma with clonally related variants differing in pulmonary metastatic potential. Clin Exp Metastasis 18: 261–271. 11315100 10.1023/a:1006767007547

[28] Kansara M, Thomas DM (2007) Molecular pathogenesis of osteosarcoma. DNA Cell Biol 26: 1–18. 17263592 10.1089/dna.2006.0505

[29] Martin BT, Kleiber K, Wixler V, Raab M, Zimmer B, et al (2007) FHL2 regulates cell cycle-dependent and doxorubicin-induced p21Cip1/Waf1 expression in breast cancer cells. Cell Cycle 6: 1779–1788. 17682292 10.4161/cc.6.14.4448

[30] Li M, Wang J, Ng SS, Chan CY, Chen AC, et al (2008) The four-and-a-half-LIM protein 2 (FHL2) is overexpressed in gliomas and associated with oncogenic activities. Glia 56: 1328–1338. 18615633 10.1002/glia.20701

[31] Tanahashi H, Tabira T (2000) Alzheimer’s disease-associated presenilin 2 interacts with DRAL, an LIM-domain protein. Hum Mol Genet 9: 2281–2289. 11001931 10.1093/oxfordjournals.hmg.a018919

[32] Labalette C, Nouet Y, Levillayer F, Colnot S, Chen J, et al (2010) Deficiency of the LIM-only protein FHL2 reduces intestinal tumorigenesis in Apc mutant mice. PLoS One 5: e10371. 20442768 10.1371/journal.pone.0010371 PMC2860980

[33] Wang J, Yang Y, Xia HH, Gu Q, Lin MC, et al (2007) Suppression of FHL2 expression induces cell differentiation and inhibits gastric and colon carcinogenesis. Gastroenterology 132: 1066–1076. 17383428 10.1053/j.gastro.2006.12.004

[34] Kinoshita M, Nakagawa T, Shimizu A, Katsuoka Y (2005) Differently regulated androgen receptor transcriptional complex in prostate cancer compared with normal prostate. Int J Urol 12: 390–397. 15948728 10.1111/j.1442-2042.2005.01093.x

[35] Muller JM, Isele U, Metzger E, Rempel A, Moser M, et al (2000) FHL2, a novel tissue-specific coactivator of the androgen receptor. EMBO J 19: 359–369. 10654935 10.1093/emboj/19.3.359 PMC305573

[36] Morlon A, Sassone-Corsi P (2003) The LIM-only protein FHL2 is a serum-inducible transcriptional coactivator of AP-1. Proc Natl Acad Sci U S A 100: 3977–3982. 12644711 10.1073/pnas.0735923100 PMC153033

[37] Purcell NH, Darwis D, Bueno OF, Muller JM, Schüle R, et al (2004) Extracellular signal-regulated kinase 2 interacts with and is negatively regulated by the LIM-only protein FHL2 in cardiomyocytes. Mol Cell Biol 24: 1081–1095. 14729955 10.1128/MCB.24.3.1081-1095.2004 PMC321437

[38] Park J, Will C, Martin B, Gullotti L, Friedrichs N, et al (2008) Deficiency in the LIM-only protein FHL2 impairs assembly of extracellular matrix proteins. FASEB J 22: 2508–2520. 18356303 10.1096/fj.07-095521

[39] Ding L, Wang Z, Yan J, Yang X, Liu A, et al (2009) Human four-and-a-half LIM family members suppress tumor cell growth through a TGF-beta-like signaling pathway. J Clin Invest 119: 349–361. 19139564 10.1172/JCI35930 PMC2631293

[40] Labalette C, Nouet Y, Sobczak-Thepot J, Armengol C, Levillayer F, et al (2008) The LIM-only protein FHL2 regulates cyclin D1 expression and cell proliferation. J Biol Chem 283: 15201–15208. 18378678 10.1074/jbc.M800708200 PMC3258904

[41] Labalette C, Renard CA, Neuveut C, Buendia MA, Wei Y (2004) Interaction and functional cooperation between the LIM protein FHL2, CBP/p300, and beta-catenin. Mol Cell Biol 24: 10689–10702. 15572674 10.1128/MCB.24.24.10689-10702.2004 PMC533999

[42] Scholl FA, McLoughlin P, Ehler E, de Giovanni C, Schafer BW (2000) DRAL is a p53-responsive gene whose four and a half LIM domain protein product induces apoptosis. J Cell Biol 151: 495–506. 11062252 10.1083/jcb.151.3.495 PMC2185594

[43] Ng CF, Ng PK, Lui VW, Li J, Chan JY, et al (2011) FHL2 exhibits anti-proliferative and anti-apoptotic activities in liver cancer cells. Cancer Lett 304: 97–106. 21377781 10.1016/j.canlet.2011.02.001

[44] Almeida M, Han L, Bellido T, Manolagas SC, Kousteni S (2005) Wnt proteins prevent apoptosis of both uncommitted osteoblast progenitors and differentiated osteoblasts by beta-catenin-dependent and -independent signaling cascades involving Src/ERK and phosphatidylinositol 3-kinase/AKT. J Biol Chem 280: 41342–41351. 16251184 10.1074/jbc.M502168200

[45] Yang Y, Hou H, Haller EM, Nicosia SV, Bai W (2005) Suppression of FOXO1 activity by FHL2 through SIRT1-mediated deacetylation. EMBO J 24: 1021–1032. 15692560 10.1038/sj.emboj.7600570 PMC554122

[46] Ambrogini E, Almeida M, Martin-Millan M, Paik JH, Depinho RA, et al (2010) FoxO-mediated defense against oxidative stress in osteoblasts is indispensable for skeletal homeostasis in mice. Cell Metab 11: 136–146. 20142101 10.1016/j.cmet.2009.12.009 PMC2819984

[47] Zhang W, Wang J, Zou B, Sardet C, Li J, et al (2011) Four and a half LIM protein 2 (FHL2) negatively regulates the transcription of E-cadherin through interaction with Snail1. Eur J Cancer 47: 121–130. 20801642 10.1016/j.ejca.2010.07.045

[48] Gullotti L, Czerwitzki J, Kirfel J, Propping P, Rahner N, et al (2011) FHL2 expression in peritumoural fibroblasts correlates with lymphatic metastasis in sporadic but not in HNPCC-associated colon cancer. Lab Invest 91: 1695–1705. 21826055 10.1038/labinvest.2011.109

[49] Kikuchi A, Yamamoto H, Sato A, Matsumoto S (2011) Wnt5a: its signalling, functions and implication in diseases. Acta Physiol (Oxf) 204: 17–33. 21518267 10.1111/j.1748-1716.2011.02294.x

[50] Wend P, Wend K, Krum SA, Miranda-Carboni GA (2012) The role of WNT10B in physiology and disease. Acta Physiol (Oxf) 204: 34–51. 21447090 10.1111/j.1748-1716.2011.02296.x

[51] Wixler V, Geerts D, Laplantine E, Westhoff D, Smyth N, et al (2000) The LIM-only protein DRAL/FHL2 binds to the cytoplasmic domain of several alpha and beta integrin chains and is recruited to adhesion complexes. J Biol Chem 275: 33669–33678. 10906324 10.1074/jbc.M002519200

[52] Fromigué O, Hamidouche Z, Marie PJ (2008) Blockade of the RhoA-JNK-c-Jun-MMP2 cascade by atorvastatin reduces osteosarcoma cell invasion. J Biol Chem 283: 30549–30556. 18757369 10.1074/jbc.M801436200 PMC2662148

[53] Fromigué O, Haÿ E, Modrowski D, Bouvet S, Jacquel A, et al (2006) RhoA GTPase inactivation by statins induces osteosarcoma cell apoptosis by inhibiting p42/p44-MAPKs-Bcl-2 signaling independently of BMP-2 and cell differentiation. Cell Death Differ 13: 1845–1856. 16470222 10.1038/sj.cdd.4401873

[54] Müller JM, Metzger E, Greschik H, Bosserhoff AK, Mercep, et al (2012) EMBO J. The transcriptional coactivator FHL2 transmits Rho signals from the cell membrane into the nucleus. EMBO J 21: 736–748. 10.1093/emboj/21.4.736 PMC125855 11847121

